# A Case of Brown Widow Envenomation in Central Florida

**DOI:** 10.7759/cureus.9165

**Published:** 2020-07-13

**Authors:** Ryan C Earwood, Jay Ladde, Philip A Giordano

**Affiliations:** 1 Department of Emergency Medicine, Florida State University College of Medicine, Tallahassee, USA; 2 Department of Emergency Medicine, Orlando Regional Medical Center, Orlando, USA

**Keywords:** brown widow, widow, spider, bite, wilderness medicine, envenomation, toxicology, insect, arachnid, antivenom

## Abstract

Latrodectus geometricus, also known as the brown widow or brown button spider, is an unrenowned relative of the American black widow. While brown widow envenomation is generally thought of as mild, it does have the potential to lead to moderate or severe features similar to black widow bites. We report a case of brown widow envenomation that led to a moderate reaction including rash, local pain, pain radiating proximally in the extremity and nausea. Poison control was consulted for aid in spider identification. The patient was treated for pain control and muscle relaxation and monitored for eight hours. After proper tetanus prophylaxis, the patient was successfully discharged home with well-controlled, but continued mild symptoms. This case highlights a little-known, but clinically relevant species of widow spider with a wide distribution. Expeditious identification and treatment of brown widow bites can increase patient comfort, satisfaction, and discharge rates.

## Introduction

Spider bites are a fairly common complaint presenting to the ED. Between 2001 and 2010, weighted annual estimates of spider bites accounted for 14.1% of all non-canine bite and sting injuries presenting to the ED [1]. Even when patients visualize a spider, or bring the spider with them, identification can be challenging. Cases in which a red or orange hourglass marking is noted on the spider can be especially concerning given the notoriety of the American black widow (*Latrodectus mactans*). Interestingly, the black widow has several lesser-known relatives, including *Latrodectus geometricus* (also known as the brown widow or brown button spider) [2,3]. The brown widow spider is found worldwide in a pantropical distribution [4]. In the United States, brown widows are found across the south from California to South Carolina and Florida [5]. While brown widow envenomation is generally considered mild, an awareness of the species, management and outcomes can aid in ED efficiency and lessen patient concern.

## Case presentation

**Figure 1:**
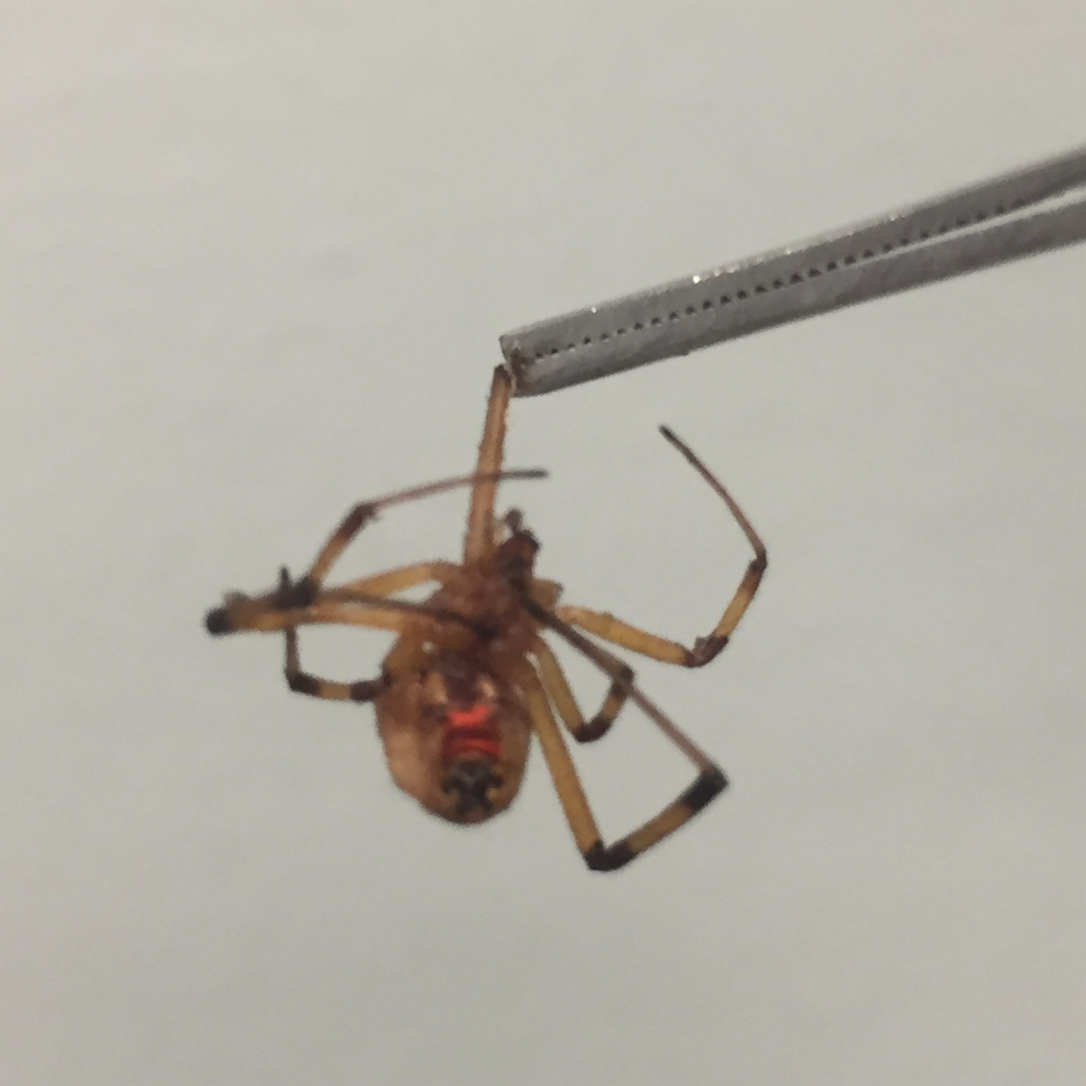
Latrodectus geometricus (brown widow) Brown widow specimen brought in by patient after bite. Note the red-orange ventral markings and banded legs, useful in identification

A 41-year-old male presented to the ED 40 minutes after suffering a spider bite to his left anterior forearm while working in his garage. The patient reported feeling immediate pain upon fang penetration and was able to kill the spider and bring it with him to the ED for identification (Figure 1). On presentation, the patient complained of nausea, a painful red circular rash at the location of the bite, and pain in the left axilla. He did not have any chest pain, shortness of breath, fever, or muscle spasm. The patient reported the pain to be a constant 6/10 on the pain scale. The pain was worse during palpation of the affected areas and relieved by raising the arm. 

**Figure 2:**
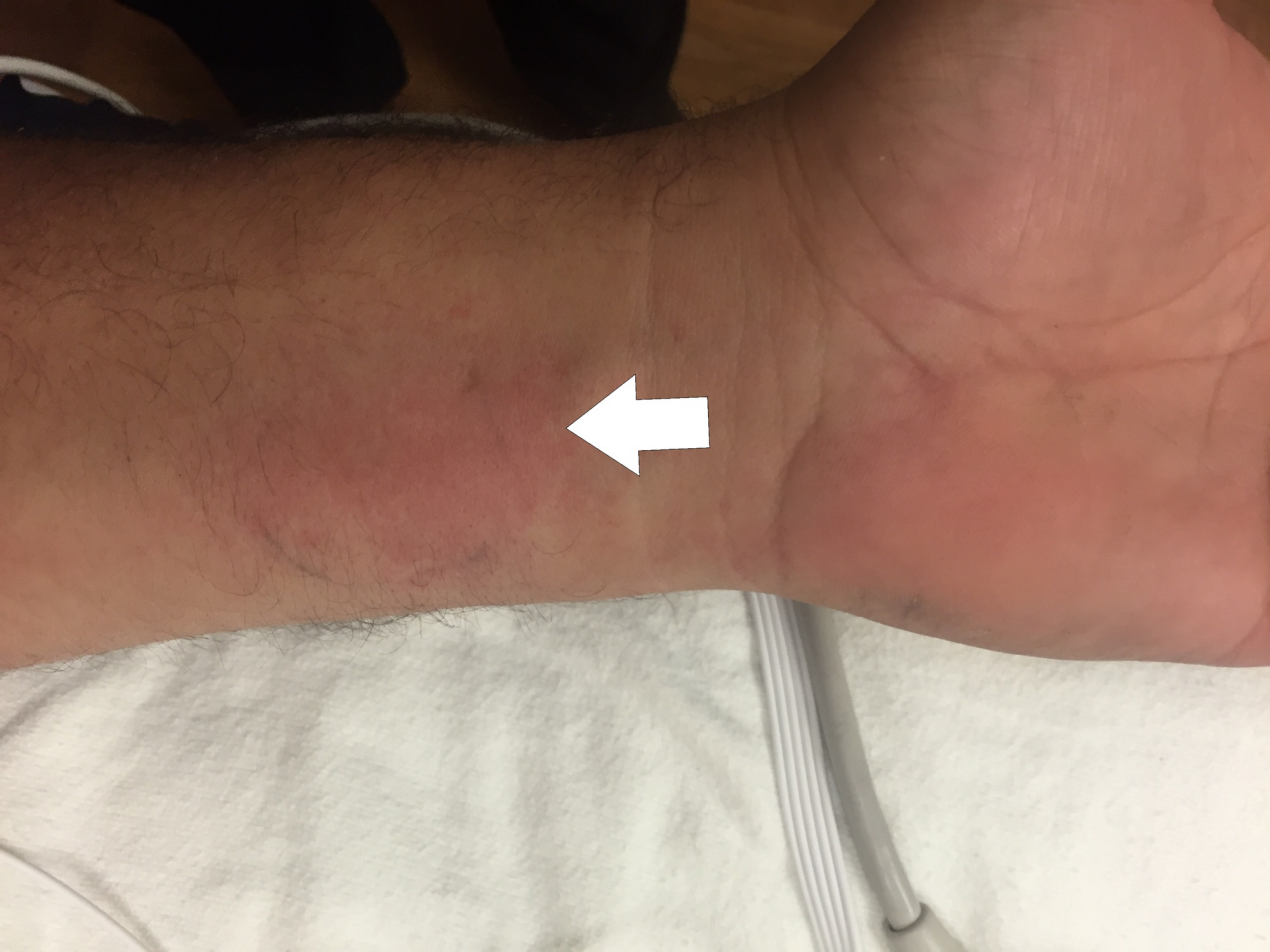
Local reaction to a brown widow bite The area of pain and erythema, marked by the white arrow, measured 6 cm in length by 3.5 cm in width

The patient’s blood pressure at presentation was 141/77 mmHg with a heart rate of 68 beats/minute. His temperature was 36.6°C (98°F), and oxygen saturation was 97% on room air. On physical examination, the patient was alert and oriented and appeared in slight discomfort. Heart examination showed a normal rate. His breathing was non-labored, and his lungs were clear to auscultation. His pupils were equal and reactive to light. Neck examination showed a supple neck without any pain. Bowel sounds were normal, and the abdomen was soft. Importantly, there was no pain or muscle rigidity provoked by abdominal palpation. Musculoskeletal examination showed a normal range of motion, but the pain was elicited with palpation of the left axilla and area surrounding the bite on the left anterior forearm. Examination of the patient’s skin showed a 6 cm by 3.5 cm area of erythema on the left anterior forearm surrounding the bite (Figure 2). There was no necrosis of the bite or surrounding skin. No laboratory evaluation was performed.

Poison control was contacted for clinical consultation and reporting. The patient was given 5 mg diazepam orally for muscle relaxation and was also given 1 mg hydromorphone IV for pain control. The patient was monitored closely over the following eight hours, watching closely for muscle rigidity, changes in pain, and tachycardia.

Roughly seven hours after presentation, the patient reported that the pain in the left arm was much improved, though he did have the continued sensation of swelling in the arm and tingling at the bite. Blood pressure at this time was 125/89 mmHg and heart rate 61 beats/minute. The patient was educated regarding the symptoms of widow bites and discharged with instructions to follow up with his primary care physician or return to the ED if worrisome symptoms develop. The patient was discharged with a three-day supply of oxycodone/acetaminophen 5 mg/325 mg tablets to be taken every six hours if needed for pain. The patient was called the next day and reported some minor pain and sent a picture demonstrating a decrease in induration and erythema. He had no further complications.

## Discussion

Widow spiders belong to the genus *Latrodectus*, which includes approximately 30 different species worldwide [2]. Not all members of this genus are implicated in human bites. A lesser-known, but still clinically relevant member of this genus is *L. geometricus* or the brown widow. Only female brown widows are capable of biting. Generally, females measure 7-10 mm in length [6]. Coloring is variable from light tan to dark brown and almost black. Darker specimens often resemble black widows; however, the ventral abdominal marking is usually yellow-orange or red-orange rather than bright red. Further, the legs of the brown widow often have banding, unlike the solid colored legs of the black widow (Figure 1). Female brown widows are not aggressive and will usually retreat unless forced against the skin [7]. Brown widows are seldom found in homes but rather living in clutter surrounding homes such as garages, woodpiles, and gardening equipment [8]. Bites most commonly occur on the extremities, usually on the arm or hand, followed by legs or foot [1].

When compared to that of the black widow, the brown widow bite is usually considered mild, characterized by pain at the penetration site and slight erythema [9]. It is important to remember that brown widows are capable of envenomation, which can lead to latrodectism via an excitatory neurotoxin known as alpha-latrotoxin [10,11]. Clinical manifestations of latrodectism include local or generalized pain, abdominal pain, diaphoresis, nausea, vomiting and autonomic effects [8]. Indeed, there have been reports of brown widow bites resulting in moderate (as seen in our patient) to severe presentations of latrodectism [7,12,13]. These rare reactions appear closer to envenomation by black widows and include severe pain, nausea, vomiting, hypertension, and fasciculations. Laboratory studies are generally non-specific and are not necessary to make the diagnosis. In severe cases, one can consider laboratory evaluation as hematuria and elevations in white blood cells, creatine phosphokinase, glucose and liver enzymes have been described [14].

Diagnosis is often made clinically, taking into account geography, history, and clinical presentation. As in our case, patients may see the spider bite them and may bring the spider with them for identification. The diagnosis in these cases can be considered definitive. In other cases, the patient may not see the cause of the bite. In these cases, if history is suspicious for a spider bite and clinical features are consistent with latrodectism, it is likely appropriate to begin treatment for a widow bite. Due to clinical features of latrodectism, including abdominal pain and chest pain, differential diagnosis in unclear cases includes non-widow arthropod bite, a surgical abdomen (appendicitis, cholecystitis), myocardial infarction, tetanus, and ectopic pregnancy [15]. In unclear cases, a work-up consistent with presenting symptoms is essential to rule out these serious pathologies.

Treatment of brown widow bites, and widow bites in general, is based on the severity of symptoms. There is little definitive evidence of symptom duration specifically related to brown widow bites, but black widow symptoms can persist up to 24 through 48 hours with symptomatic treatment alone [14]. In mild cases (pain and erythema localized to the bite without migration or systemic symptoms), the wound can be cleaned, and tetanus prophylaxis can be provided if indicated. Oral analgesics such as ibuprofen or oxycodone/acetaminophen can be used to alleviate the pain associated with the bite. Oral benzodiazepines (diazepam) or methocarbamol have been used for muscle relaxation, but there is evidence of increased adverse events associated with the use of benzodiazepines [16]. As the severity of symptoms increases, so does the level of supportive care. In moderate to severe cases (radiation of pain, nausea, and true latrodectism), wound care and tetanus prophylaxis are still suggested. Pain management can be administered parenterally (morphine, hydromorphone), as can muscle relaxants (lorazepam). Finally, in cases resistant to supportive therapy, consideration can be given to the use of antivenom. There are several commercial antivenoms available, and the use of antivenom has been shown to decrease pain duration and severity such that discharge home is more likely [14,17]. Adverse events associated with the use of antivenom include anaphylaxis, serum sickness and rarely death [14,17-19]. As such, it is highly suggested that consultation with a clinical toxicologist or another widow bite expert is carried out. Due to the low availability of antivenom in the United States and possible risks associated with its administration, clinicians should call their regional poison control center at 1-800-222-1222 for guidance.

## Conclusions

Spider bites are a fairly common concern in the ED. Familiarity with the clinical spectrum of latrodectism will help guide clinicians in treatment when patients present with these concerning symptoms. Further familiarity with less common species of spiders, such as the brown widow, can also aid in the efficiency of identification and lead to quicker treatment and less patient stress. To date, there have been very few reports of brown widow envenomation; however, there is evidence that brown widows can cause severe latrodectism. This report describes a moderate reaction to a brown widow bite and successful treatment strategies.
